# Housing Affordability, Housing Tenure Status and Household Density: Are Housing Characteristics Associated with Union Dissolution?

**DOI:** 10.1007/s10680-019-09549-6

**Published:** 2020-01-10

**Authors:** Sandra Krapf, Michael Wagner

**Affiliations:** 1 Mannheim Center for European Social Research, University of Mannheim, 68159 Mannheim, Germany; 2 Institute of Sociology and Social Psychology, University of Cologne, Albertus-Magnus-Platz, 50923 Cologne, Germany

**Keywords:** Housing cost, Household crowding, Homeownership, Separation, Socioeconomic, Situation

## Abstract

Housing is an important dimension of social inequality between couples, but it has been largely ignored in prior research on union dissolution. Extending the literature that controlled for the stabilizing effect of homeownership, we investigate whether housing, measured as household density, housing tenure and housing affordability, is related to the risk of union dissolution. Based on data from the German Family Panel (pairfam), we analyze 3441 coresidential partnerships. We run discrete-time event-history models to assess the risk of separation within a time frame of 7 years. Housing affordability is found to be negatively related to the risk of union dissolution among couples, as those couples with a high residual income (i.e., household income after deducting housing costs) were less likely to separate than those with a lower residual income. By contrast, household density is found to be unrelated to separation. In line with previous research, our findings indicate that homeowners had more stable relationships than tenants. The analysis shows that this was the case regardless of whether the home was jointly owned or was owned by one partner only.

## Introduction

Partnership dissolution is a widespread phenomenon in advanced societies. Such breakups can have negative consequences for both the partners (Andreß and Bröckel 2007; Kalmijn 2010) and their children (Amato and James 2010). Therefore, it is important to understand whether the risk of separating is unequally distributed across specific socioeconomic groups. The existing empirical evidence has, however, been inconclusive (Lyngstad and Jalovaara 2010; Killewald 2016; Wagner and Weiß 2003). In this paper, we focus on an important dimension of the socioeconomic situations of couples, namely their housing conditions. Given the social stratification of housing outcomes (Kurz and Blossfeld 2004; Dewilde and De Decker 2016; Zavisca and Gerber 2016) and the centrality of housing for couples’ everyday lives, it seems surprising that, apart from studies that include homeownership as a control variable in statistical models, there has been little research explicitly focusing on the association between housing conditions and relationship stability. We investigate to what extent housing tenure (with joint and sole homeownership as distinct categories), housing affordability and household density are associated with the risk of union dissolution.

Housing characteristics are closely related to the overall socioeconomic situations of couples, as wealth is positively associated with homeownership, home size and housing affordability. We argue, however, that housing has an independent net influence on the risk of union dissolution because our three indicators are determined not only by couples’ income levels, but by the housing market conditions in their local areas. If house prices increase, a smaller share of the local population will have access to homeownership, unless earnings levels and/or interest rates also adapt. Similarly, household density and affordability depend on the availability of spacious and affordably priced housing. From this perspective, analyzing the impact of housing characteristics on the risk of union dissolution is important because, in contrast to other dimensions of the socioeconomic status of a couple, housing is affected by specific external conditions, including the available housing stock, house prices, the state of the rental market and access to mortgages. This is of special interest in the context of changing housing markets—and although the German housing market has been comparably stable for years, house prices rose rapidly in the past decade (Wijburg and Aalbers 2017).

In the following chapter, we discuss prior research findings. Section 3 is devoted to our theoretical considerations. Our empirical analyses focus on the association between the risk of union dissolution on the one hand; and housing affordability, household density, as well as a refined measure of housing tenure on the other. We use data from the German Panel Analysis of Intimate Relationships and Family Dynamics (pairfam) for the years 2008 to 2015 (see Sect. 4). The results of our discrete-time event-history models are presented in Sect. 5. The last section concludes.

## Previous Research

A large number of previous studies have examined the determinants of union dissolution, including couples’ employment situations, income levels, education and demographic situations (Lyngstad and Jalovaara 2010; Amato 2010; Härkönen 2014). Many of these studies controlled for housing tenure in their statistical analysis of union dissolution patterns. Although a few authors found no statistically significant association between homeownership and the likelihood of union dissolution in specific sub-groups (Yabiku et al. 2009; Cooke 2006 for the western German subsample; Killewald 2016 for the marriage cohorts of 1968–1974 in the USA; Coulter and Thomas 2019 found significantly increased separation risks only among social renters compared to homeowners, but not among private renters in the UK), a majority found that homeowners have more stable relationships than tenants (Grinstein-Weiss et al. 2014; Wagner and Weiß 2003; Bracher et al. 1993; South 2001; Wiik et al. 2009; Kaplan and Stier 2017; Jalovaara 2002; Ostermeier and Blossfeld 1998; Kaplan and Herbst 2015; Raz-Yurovich 2012). However, the associations between union dissolution and other factors related to housing, such as household density and affordability, have been studied less often (but see Coulter and Thomas 2019 for an exception).

The existing evidence on crowding in the household and union dissolution is mixed. A study for Russia found that the number of rooms in a dwelling was negatively related to the probability of divorce (Gerber and Zavisca 2015). Also, Coulter and Thomas (2019) found evidence that space pressure was positively related to separation in the UK. In Finland, Jalovaara (2002) found that living in overcrowded housing conditions was associated with elevated divorce risks, but that this result was not robust after controlling for other socioeconomic factors. Moreover, a recent study in Luxembourg showed that the association between crowding and union dissolution disappeared after controlling for homeownership (van Damme 2019). O'Connor et al. (1999) found a bivariate relationship between crowding and separation in England, but also that this relationship vanished after further covariates were taken into account.

With a focus on Sweden, one study has examined housing affordability and its association with the risk of divorce at the micro-level (Lauster 2008). The results showed that the ability to cover housing payments was positively related to partnership stability, but only among renter households. Among homeowners, no association was found between housing cost burden and partnership stability. Additionally, a recent study of the UK found that mortgage or rent arrears were associated with an increased union dissolution risk (Coulter and Thomas 2019).

A number of other studies that focused on the link between socioeconomic status and partnership quality in general found that economic hardship is negatively related to partnership quality (Hardie et al. 2014; Conger et al. 1990, 2010). However, the empirical evidence on the relationship between financial resources and partnership stability is less clear: some studies that examined this issue found no statistically significant association between household income and the risk of union dissolution (Killewald 2016; Schoen et al. 2002; Cooke 2006), whereas others found that couples with lower incomes have higher dissolution risks (Brines and Joyner 1999; Dechter 1992; Kalmijn et al. 2007; Kaplan and Herbst 2015; Raz-Yurovich 2012; Jalovaara 2013). Many of these studies distinguished between the effects of husbands’ and wives’ socioeconomic resources. While husbands’ resources were shown to be associated with higher levels of union stability (Lyngstad and Jalovaara 2010), the findings for wives’ earnings and labor force participation were rather mixed (see Härkönen 2014; Özcan and Breen 2012 for recent reviews). In summary, most previous studies find a stabilizing effect of homeownership on partnerships. The few analyses on the effect of housing affordability indicate that housing costs are positively associated with union dissolution, while there is no clear pattern for the effect of household density.

## Theoretical Background: Housing and Union Dissolution

In analyses that seek to explain the dissolution of unions at the micro-level, ideas from exchange theory (Rusbult 1980; Lewis and Spanier 1979; Levinger 1979) and the New Household Economics (e.g., Becker et al. 1977) are usually employed. Both strands of this theoretical literature suggest that partnerships end because in the long run, their (material and non-material) costs exceed their (material and non-material) benefits for at least one partner. It therefore appears likely that the anticipated costs of separation affect the decision to dissolve or to remain in a union.

One potential barrier to separation is homeownership. The purchase of a home is generally seen as an investment in a relationship. Homeownership is, moreover, closely related to a household’s economic resources. As buying a house is usually the largest investment a household makes, the home is often the most important component of a family’s wealth. After a breakup, the value of the investment in joint property depreciates for at least one partner, either because only one partner can stay in the house, or because selling the house produces transaction costs. Thus, unions of homeowners tend to be more stable than those of tenants.

Additionally, it has been argued that the homeownership effect might be a result of a selection process, whereby the partners anticipate the negative consequences of separation for their housing situation and therefore invest in ownership only if they expect to remain together (Lersch and Vidal 2014). In line with this selection argument, we assume that couples who are happier in their relationship are more willing to increase their level of commitment by purchasing a house together. At the same time, such couples are also less likely to break up (Brüderl and Kalter 2001).

Apart from increased relationship commitment, homeownership is related to higher housing quality. The housing conditions of homeowners tend to be better than those of tenants (Mulder and Smits 1999). On average in Germany, owner-occupied houses and apartments are larger, have more rooms and are newer than rented dwellings (DESTATIS 2013). Assuming the household has sufficient resources to cover the costs of homeownership, couples who own a home tend to have more opportunities than tenants for personal fulfillment, as they can create a comfortable home that corresponds to their individual preferences. This freedom might be associated with higher levels of overall satisfaction, which may in turn be linked to increased relationship quality and relationship stability.

Based on these considerations, we expect to find that couples who live in a home they own have lower union dissolution risks than those who are tenants (H1a). It has long been argued that homeownership represents a partnership-specific investment by a couple. However, recent studies have pointed out that some couples live in a house that is owned by only one of the partners (Lersch and Vidal 2016) or by a third person, such as one of the partner’s parents or other kin (Gerber and Zavisca 2015); and that the dissolution of such partnerships would not involve the depreciation of a joint investment. We therefore expect to find that couples who live in a home that is owned by only one of the partners or by a third party (e.g., one partner’s parents) have higher separation risks than joint homeowners (H1b).

In the case of sole homeownership, the effect of homeownership on union stability may vary according to the gender of the homeowner. If the female partner is a sole homeowner, this might be a sign of her economic independence, one factor that enables women to leave an unhappy relationship (Sayer et al. 2011). By contrast, a male partner’s ownership might stabilize the partnership because it is in line with the gender norm that men should provide for the family. A recent study from the UK provides some support for this gendered resources argument as it relates to housing (Coulter and Thomas 2019). The authors investigated the association between who is written into the dwelling contract (rental or ownership) and separation risks. Their analyses showed that separation was more likely when only the woman was a contract holder, as compared to when both partners or only the man held contractual rights. Similarly, we expect that if the woman is the sole homeowner, a couple has a higher separation risk compared to joint homeowners (H1c). For male sole homeowners, we do not expect such a difference.

Another housing-related factor that may affect union stability is household density. Partners who live in overcrowded housing might have increased stress levels and be dissatisfied with their lifestyle. This dissatisfaction may in turn be associated with reduced partnership quality (Lewis and Spanier 1979) and an increased risk of union dissolution. Household crowding refers to a lack of space in the home of an individual or a family and is typically defined as a situation in which there is more than one person per room in the home (Gove and Hughes 1983). To ensure that the people living in a household have the opportunity to withdraw from social interactions, the home must have a sufficient number of rooms to allow each individual member to spend some time alone. In the social psychology literature, it has been shown that individuals experience psychological distress in crowded living conditions (Evans 2003; Wells and Harris 2007). If one partner is exposed to stress, the other partner can support him or her in dealing with the challenging situation (Bodenmann and Cina 2006). If, however, household density is high, both partners are subject to the stress-creating situation and might have reduced coping capacities. Although dyadic coping is largely mediated through communication, a high level of density seems to reduce the ability of individuals to interact socially. It has, for example, been shown that crowding can lead to withdrawal (Evans and Lepore 1993), depression, or aggression (Regoeczi 2008). As these behaviors or emotional states are negatively associated with partnership quality (Roberts 2000), it is likely that they also negatively affect partnership stability. Based on these considerations, we expect to find that couples who live in a household with a high density level face a higher risk of union dissolution than couples who live in a household with a low density level (H2a).

Rather than separating, a couple may seek to escape a crowded housing situation by moving to a more spacious dwelling. Residential mobility is a means of adjusting the family’s housing situation to the needs of the members (Rossi 1955; Wagner and Mulder 2015). For example, families may move before or after the birth of a child because they need (or anticipate the need for) more space (Kulu 2008). Couples who can afford to move to a larger apartment will generally choose to avoid crowded living conditions. However, couples of low socioeconomic status might have limited financial means to move to a larger home because rents and/or search costs are high. For these couples, high-density living may be a financial necessity. Thus, they may be exposed to suboptimal conditions for a longer time than are couples who can afford to move to a more spacious dwelling. We hypothesize that living in a household with a high level of density is especially detrimental to partnership stability if a couple has a high housing cost burden (H2b).

Our third hypothesis focuses on the cost of housing. Housing affordability refers to the share of the available income of a household that is used to cover housing costs. These costs include all of the expenditures related to housing consumption, such as rent, loan repayments and utilities. For many individuals and families, housing costs take up a large share of their household budget and represent their single largest monthly expenditure (OECD 2011; Boehm and Schlottmann 2008). The possible associations of housing costs and union dissolution are complex and lead us to two contradictory arguments. On the one hand, high housing costs can lead to psychological distress of each partner and thus increase the risk of union dissolution. Financial worries might create individual stress, which can in turn lead to reduced coping abilities and negative interaction patterns between partners. Existing studies have found that individuals suffer more from housing cost burdens than from financial strain in general (Taylor et al. 2007). One potential explanation for this finding is that because housing is a basic need, housing costs usually cannot be reduced by simply adjusting consumption levels. This is particularly likely to be the case in tight housing markets. In such a situation, a high housing cost burden might limit a couple’s ability to pay for other essential needs. Based on the assumption that stress leads to lower relationship quality and decreased union stability, we expect to find that couples who have only a little money left over after housing costs are deducted, i.e., who face a higher housing cost burden, are more likely to break up (H3a).

On the other hand, taking an exchange theoretical perspective, the association between (regional level) housing affordability and union dissolution might be positive. Lewis and Spanier (1979) suggest that a low evaluation of alternatives prevents an individual from separation. Translated to the housing situation, a person who wants to split up with his coresidential partner might have difficulties finding affordable housing and therefore may choose not to separate. Unfortunately, our data do not include explicit information about the availability of affordable and suitable housing for individuals following a separation. However, the data do include settlement size. Because house prices are generally higher in urban than in rural areas of Germany (Roos 2006), we assume that it is generally easier to find an affordable dwelling after separation in smaller communities compared to big cities. Following from this idea, we expect that the effect of housing affordability on union dissolution depends on the area, such that a person who lives in an urban area (especially one with a higher housing cost burden) has greater difficulties in finding affordable housing after separation than someone living in a rural area. Therefore, we expect that among couples with a high housing cost burden, those living in urban areas will have a lower risk of union dissolution than those living in rural areas (H3b).

## Data and Methods

Our analyses are based on a longitudinal dataset, the German Family Panel pairfam (Brüderl et al. 2017; Huinink et al. 2011) covering the period between 2008/09 (Wave 1) and 2014/15 (Wave 7). The dataset includes the partnership histories of three birth cohorts (born in 1991–1993, 1981–1983 and 1971–1973). Given our focus on coresidential unions, we have analyzed couples in which the partners were at least 18 years old and were living together in a household. In order to be able to analyze the potentially gendered nature of sole homeownership (hypothesis H1c), we dropped same-sex couples from our analyses. The information on each main respondent’s housing situation was collected at the time of the interview, not in a retrospective manner. Therefore, we reduced the sample to include only those couples who reported being in a coresidential partnership at the time of the interview.

Unfortunately, as is the case for many other social survey datasets, information on housing costs and/or household income is missing for a relatively large share of the respondents (32%) in our sample. Ignoring missing values by exclusively analyzing complete cases might lead to inefficient and/or biased estimates in multiple regression analyses (Meinfelder 2014; van Buuren 2012). To deal with this problem, we imputed the missing income and housing cost information using Multiple Imputation by Chained Equations (MICE). We used the predictive mean matching algorithm to multiply impute values for the income and the housing cost variables based on longitudinal data from the three nearest neighbors (van Buuren 2012). Using Stata 15, we created 20 imputed datasets. The multiple imputation of income and housing cost reduced the share of missing observations from 32 to 12%.

To determine the association between housing conditions and separation risks, we used a discrete-time hazard model for the transition to union dissolution. The data were organized in a discrete-time event-history format, with relationship-years as the unit of observation. The process time was the duration of the coresidential union. Assuming that the underlying latent time variable was continuous, we specified a complementary log–log model (Allison 1982). To adjust for multiple observations of individuals in the period under study, we specified the panel robust standard errors.^1^ The information on the start of coresidence and on the time of union dissolution was based on the dates reported by the respondents. All variables in our analyses are time-varying. Using the information on housing conditions and all other variables at the time of the interview, we analyzed the risk of union dissolution in the following waves (i.e., all explanatory variables were lagged by 1 year). Using the imputed data, we analyzed data from 3441 coresidential couples (16,036 relationship-years). The separation event was identified by the end date of coresidence in a partnership which was available in pairfam’s relationship histories. In addition, the data include information about the end date of the relationship. To ensure that we were analyzing only those respondents who ended their intimate relationship, and did not simply stop living together (e.g., couples who moved into two separate households for work-related reasons), we excluded couples who did not dissolve their union within 3 months after one partner moved out of the joint household.^2^ Most of the relationships in our sample were stable over the study period, but there were 401 cases in which the relationship ended (2.5% of the relationship-years or 11.7% of the couples).

### Measuring Housing Characteristics

Our first housing characteristic is *housing tenure*. The homeownership rate in Germany in 2013 was 43% (DESTATIS 2013). It should be noted that Germany has one of the lowest homeownership rates in Europe (Mulder and Billari 2010). The median age at entry into first homeownership strongly depends on the birth cohort (Wagner and Mulder 2000), and the average age at entry into homeownership in Germany is around the mid-thirties (Angelini et al. 2013). In most of the studies that have been conducted on this topic, the available information on housing tenure did not allow the researchers to distinguish between different types of owners. Some of these studies focused on comparing joint homeownership with all other housing arrangements (Brüderl and Kalter 2001; Rapp et al. 2015), whereas others took into account whether the property was inherited (Ostermeier and Blossfeld 1998). Only some recent studies analyzed sole homeownership (Lersch and Vidal 2016; Eads and Tach 2016; Coulter and Thomas 2019); and, to our knowledge, just one of these studies identified non-tenants who were living in a third party’s property (Gerber and Zavisca 2015). In our analyses, we distinguish between four groups: (1) couples who were joint homeowners, (2) couples in which only one of the partners was a homeowner, (3) couples who were tenants and (4) others (e.g., couples who were living in a property that belonged to their parents or a third person, but who were not tenants). As we can see in Table 1, more than half of the respondents in our sample were living in a rented dwelling, 26.6% were living in a jointly owned property, 15.7% were living in a dwelling that was owned by only one of the partners and 5.0% belonged to the *other non-tenant* group. In order to test the gendered effect of sole homeownership (hypothesis H1c), we also distinguished between male (9.6%) and female homeowners (6.0%).

**Table 1:** Descriptive statistics of the analytical sample. Means and column percent of relationship years (complete cases). *Source*: Pairfam waves 1–7. Authors’ own calculation. Means and percentages refer to data before multiple imputation

Duration of coresidential union in years	Mean: 8.9
Duration of coresidential union in years squared	Mean: 111.2
Age of female partner	Mean: 33.4
Children living in the household	
No children	26.5
1 Child	26.6
2 Children	33.3
3 Or more children	13.7
Partnership status	
Unmarried	27.5
Married	72.5
Region	
Western Germany	78.9
Eastern Germany	21.1
Housing tenure	
Joint homeownership	26.6
Sole homeownership	15.7
Tenants	52.7
Other (e.g., living in parents’ property)	5.0
Household density	
Low (< 1 person/room)	80.4
Medium (1 person/room)	13.6
High (> 1 person/room)	6.0
Housing affordability	
Low residual income	31.7
Medium residual income	34.8
High residual income	33.6
Number of relationship-years (complete cases)	14,062
Number of separations (complete cases)	371

Another key variable in this study is *household density*. In the literature, several different objective indicators of density and (over)crowding on the household level have been suggested. The term crowding generally refers to an individual’s subjective perception of having a lack of space (Stokols 1972). Because the pairfam data do not include information on such subjective measures, we use the number of rooms and persons to calculate an objective measure of household density. Building on studies in the housing and psychology literature, we define household density as the ratio of persons per room (Clark et al. 2000; Gove and Hughes 1983; Conley 2001; Gómez-Jacinto and Hombrados-Mendieta 2002; Regoeczi 2008; Evans 2003). In pairfam, the question about the number of rooms was posed as follows: *And how many rooms does this apartment (or this house) have?* If the respondent found this question unclear, the interviewer instructed her or him to count only the rooms that are larger than six square meters, and that are not bathrooms or kitchens. Thus, in our analysis, the number of rooms refers to the number of bedrooms, living rooms and other rooms, such as workrooms. Based on the responses to this question, we calculated the ratio of individuals living in the household to the crude number of rooms. Clearly, space demands vary across individuals but pairfam data do not include details about such differences. However, in line with other studies, we assume that children need less space than adults (e.g., Lersch 2014; Rybkowska and Schneider 2011). Therefore, in our person–room ratio, we count children under age 12 as 0.5 of a person, and children aged 12 or older as a full person.^3^ The smaller the value of the indicator, the lower the level of household density (or, in other words, the more spacious the dwelling). Conversely, a higher value indicates a higher level of density, and thus less space in the dwelling. In the literature, a number of studies have dichotomized this variable and defined (over-)crowding as referring to more than one person per room. In our study, we decided to use three categories: less than one person per room, one person per room and more than one person per room on average. This system of categorization allows us to identify whether the threshold of *less than one person per room* is informative for our analyses. Table 1 reveals that 6.0% of respondents in our sample were living in a high-density household.^4^ Another measure of crowding could differentiate between the statuses of the household members. For example, it may be seen as less detrimental for the partners’ relationship if they are sharing a crowded household with their own children, rather than with other people. However, since in our sample less than 3% of couples reported sharing a household with other people, this category appears to be less relevant in the German setting.

Our third housing characteristic is *housing affordability* on the household level*.* In analyzing this variable, we followed the residual income approach (Stone 2006; Haffner and Heylen 2011), in which the amount of income left over after housing costs are deducted is divided by the number of household members. Many of the previous studies that looked at the impact of affordability used the share of housing costs in the monthly household income in their analyses. In Germany, the housing cost overburden rate is 23.6%; i.e., in nearly one-quarter of German households, the total housing costs exceed 40% of the household income (Rybkowska and Schneider 2011). However, focusing on the share of income spent on housing might be misleading, as rich households can afford to spend a larger percentage of their income on housing than poor households (Stone 2006). Because we are interested in the level of the stress couples feel as a result of high housing costs, we use the residual income measure on the individual level. Couples are especially likely to struggle to cover their housing costs if their residual income after paying for housing is low. If the partners have to negotiate even small expenditures, their psychological distress and fights about financial issues are likely to increase.

Our calculation of the residual income follows the suggestions made in Haffner and Heylen (2011). To determine the housing costs of owner occupiers, we used the information on monthly expenditures on utilities (heat, electricity, water) and on mortgages or building loans. To determine the housing costs of tenants, we used monthly expenditures, including rent and utilities. These items were part of the pairfam question program in Waves 1, 3 and 5 only. For Waves 2, 4 and 6, we used the values reported in the previous wave, but only if the respondent had not moved to a new place in the meantime. In Waves 3 and 5, the respondents were asked the question only if they had reported a residential move. This approach was based on the assumption that each couple’s housing costs changed relatively little in subsequent years unless they had moved to a new dwelling. The reported amount was then deducted from the monthly net household income.

The information on household income might be biased, as the respondents might, for example, have systematically reported having an income that was higher or a lower income than their actual income. The pairfam team checked for such measurement errors by validating the incomes reported in pairfam with the incomes reported in the German Socio-Economic Panel (SOEP), which is regarded as a high-quality dataset. This validation showed that the results in the two datasets were almost identical (Arránz Becker et al. 2013). Based on the assumption that the income figures reported in the SOEP are correct, we can conclude that the income data reported in the pairfam are unbiased.

To account for the household structure, we divided the amount of income by the number of household members. Unfortunately, we do not have detailed information about consumption patterns in couples. Inspired by the calculation of equivalized disposable household income, we included each child under age 12 as 0.5 of a person (instead of as a full person) because children on average might consume less than adults. The affordability measure was broken down into three categories based on income terciles: a low-, a medium- and a high-residual-income group. Our measure of housing affordability was found to be closely related to household income: The absolute housing costs (including rent and utilities for renters and utilities and mortgages for owners) and the net household income in euros were found to be correlated with *R* = 0.33.^5^ Although this is a moderate statistical association, the value implies that housing costs and income are not completely collinear.

Our measure of housing affordability ignores that the residential choices depend not necessarily only on cost aspects but also on lifestyle preferences (Ærø 2006). Unfortunately, the pairfam data do not include information on housing preferences. There is also no explicit measurement of housing affordability at the regional level in the data. In order to test hypothesis H3b, we use the size of the settlement where a respondent lives at time of interview as a proxy measure for the affordability of the local housing market. In order to identify potentially nonlinear associations, we distinguish between small (< 20,000 inhabitants), medium (20,000 to 100,000 inhabitants) and large settlements (> 100,000 inhabitants).

### Other Covariates

In the multiple regression models, we controlled for the possible confounding effects of a number of factors. The descriptive statistics for these variables can be found in Table 1. The process time was the *duration of the coresidential union* at the time of the interview. We included in the model duration in months and duration in months squared. We also controlled for *age of the female partner* (because we did not expect any different effects for the male partner’s age, we chose not to add it to the models). Furthermore, we included the *number of children* in the household in our multiple regression analyses. The more children there were, the higher the household density was, and the lower the residual income was after housing costs were deducted. From a theoretical perspective, having children can be seen as investing in a partnership. Indeed, empirical research has shown that having children is a barrier to union dissolution. We can therefore assume that parenthood is a stabilizing factor in a relationship (Lyngstad and Jalovaara 2010; Wagner and Weiß 2003). In the sample, 26.5% of the couples had no children living in the household, 26.6% were living with one child, 33.3% were living with two children, and 13.7% were living with three or more children. Another control variable we included was *marital status*. It has been argued that married couples are more committed to their partnership than couples who share a household without being married (Stanley et al. 2004). In the data, the vast majority (72.5%) of the couples were married. (If a couple married in the course of the seven panel waves, the time-varying variable *marital status* was changed from unmarried to married.) We also accounted for *regional differences* using a binary variable denoting residence in either western or eastern Germany. It has been shown that in the decades since the fall of the Berlin Wall, homeownership has been less common in eastern than in western Germany (Frick and Grimm 2010), and divorce rates have been lower in the east than in the west (Grünheid 2013). More than three-quarters (78.9%) of our respondents were living in western Germany.

In sensitivity analyses, we used two additional control variables: relationship satisfaction and net household income. We measured relationship satisfaction via the following question: *Overall, how satisfied are you with your relationship?* The response scale ranged from zero (*very unsatisfied*) to 10 (*very satisfied*), and the responses in our sample were strongly skewed, with a mean value of 7.9. The categorization of the satisfaction variable along the terciles thus led to a larger *high satisfaction* group (48.1%). Just 23.6% of the respondents were categorized as having a medium level of satisfaction, and 28.3% were categorized as having a low level of satisfaction. Like the other variables, the satisfaction variable was measured in the wave before the separation information was reported. The *net household income* variable measured post-tax monthly income in 100-euro increments.^6^

## Multiple Regression Results

**Fig. 1:**
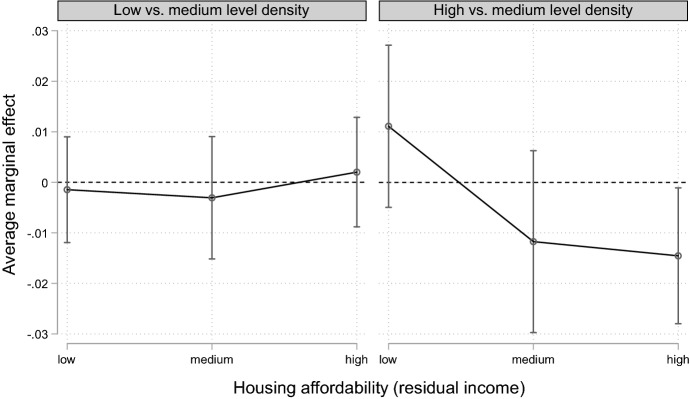
Interaction effect (Model 6). Average marginal effects of household density on union dissolution for different levels of housing affordability (residual income after housing costs are deducted). Reference category: medium-level density (dashed line). *Source*: Pairfam waves 1–7. Authors’ own calculation. Multiply imputed data. Control variables: duration of coresidential union, duration squared, age of female partner, partnership status, region, number of children in the household, housing tenure. Average marginal effects and significance levels are presented in Table 6 in “Appendix”

**Fig. 2:**
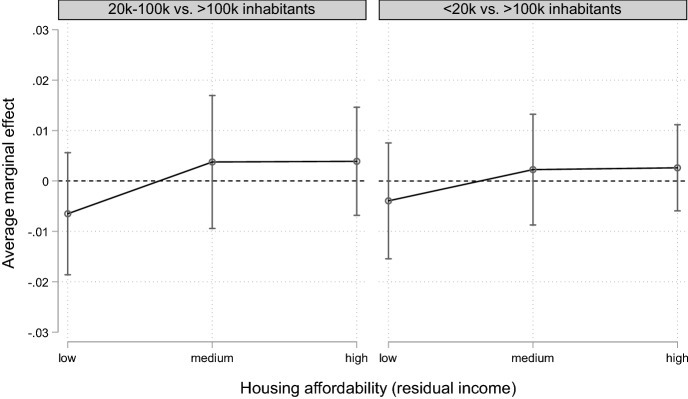
Interaction effect (Model 7). Average marginal effects of settlement size on union dissolution for different incomes (residual income after housing costs are deducted). Reference category: large settlement size (> 100,000 inhabitants, dashed line). Source: pairfam Waves 1–7. Authors’ own calculation. Multiply imputed data. Control variables: duration of coresidential union, duration squared, age of female partner, partnership status, region, number of children in the household, housing tenure, household density. Average marginal effects and significance levels are presented in Table 6 in “Appendix”

The results of the discrete-time event-history analyses based on the multiply imputed data are shown in Table 2 and Figs. 1 and 2. In Table 3, we present the results of our sensitivity analyses. The results of the multiple regression analyses on the basis of the complete cases analyses (i.e., without imputation) are provided in Table 4 in “Appendix”. The sizes of the average marginal effects (AMEs) of the complete cases analyses were largely in line with those based on the multiply imputed data, although the significance levels differed for a number of coefficients. The dependent variable is the transition to separation; in our case, the dissolution of coresidential partnerships. We present the average marginal effects, as they allow us to compare effect sizes across different models (Mood 2010). The average marginal effect in a logistic regression depends on the value of all of the explanatory variables; it is the mean of the marginal effects for each combination of covariates in the dataset and represents the average change in the probability of observing a specific outcome when we alter the respective independent variable from the reference to a different category based on our sample. In Table 5, we additionally present the results of the main analyses as odds ratios.

**Table 2:** Discrete-time event-history models of union dissolution (average marginal effects). *Source*: Pairfam waves 1–7. Authors’ own calculation

	Model 1	Model 2	Model 3	Model 4	Model 5
Duration of coresidential union					
In years	− 0.001	− 0.001	− 0.001	− 0.001*	− 0.001
In years squared	0.0001***	0.0001***	0.000*	0.0001*	0.0001**
Age of female partner	− 0.001***	− 0.001***	− 0.001**	− 0.001***	− 0.001***
Children living in the household					
No children	0.008*	0.008*	0.010**	0.007***	0.010**
1 Child	0	0	0	0	0
2 Children	0.005	0.005	0.003	0.004	0.004
3 Or more children	0.005	0.005	0.003	0.003	0.004
Partnership status					
Unmarried	0	0	0	0	0
Married	− 0.022**	− 0.022**	− 0.025**	− 0.025***	− 0.022**
Region					
Western Germany	0	0	0	0	0
Eastern Germany	0.006*	0.006*	0.006^#^	0.006**	0.005^#^
Housing tenure					
Joint homeownership	0				0
Sole homeownership	0.004				0.004
Tenants	0.013**				0.013**
Other (e.g., living in parents’ property)	0.024**				0.024**
Housing tenure					
Joint homeownership		0			
Male sole homeowner		0.005			
Female sole homeowner		0.002			
Tenants		0.013**			
Other (e.g., living in parents’ property)		0.024**			
Housing affordability					
Low residual income			0.000		− 0.001
Medium residual income			0		0
High residual income			− 0.007*		− 0.007*
Household density					
Low (< 1 persons/room)				− 0.002	0.001
Medium (1 person/room)				0	0
High (> 1 person/room)				0.004	0.002
Number of relationship-years Number of events	16,036 401	16,036 401	16,036 401	16,036 401	16,036 401

^#^*p* < 0.1, *p < 0.05, **p < 0.01, ***p < 0.001. Multiply imputed data

**Table 3:** Sensitivity analyses. Discrete-time event-history models of union dissolution (average marginal effects). *Source*: Pairfam waves 1–7. Authors’ own calculation

	Model 8 Only couples with partnership duration < 6 years	Model 9 Including control variable *partnership satisfaction*	Model 10 Including control variable *absolute income*
Duration of coresidential union			
In years	0.021**	− 0.001	− 0.001
In years squared	− 0.004**	0.0001*	0.0001*
Age of female partner	− 0.002^**^	− 0.001^**^	− 0.001^**^
Children living in the household			
No children	0.019**	0.014**	0.010**
1 Child	0	0	0
2 Children	0.016	0.003	0.004
3 Or more children	0.021	0.004	0.003
Partnership status			
Unmarried	0	0	0
Married	− 0.030**	− 0.017**	− 0.022**
Region			
Western Germany	0	0	0
Eastern Germany	0.003	0.006^*^	0.005^#^
Partnership satisfaction			
Low		0.032**	
Medium		0	
High		− 0.008**	
Housing tenure			
Joint homeownership	0	0	0
Sole homeownership	0.030^#^	0.002	0.004
Tenants	0.038**	0.012**	0.013**
Other (e.g., living in parents’ property)	0.056**	0.019**	0.024**
Housing affordability			
Low residual income	0.014**	− 0.001	− 0.001
Medium residual income	0	0	0
High residual income	− 0.005	− 0.005^#^	− 0.007*
Household density			
Low (< 1 persons/room)	− 0.000	0.001	0.001
Medium (1 person/room)	0	0	0
High (> 1 person/room)	0.003	0.002	0.002
Net household income in 100 euros			0.00003
Number of relationship-years	5091	15,934	16,036

^#^*p* < 0.1, *p < 0.05, **p < 0.01, ***p < 0.001. Multiply imputed data

### Main Analyses

In our main analyses, we ran seven different models. In Models 1 to 4, we included each housing variable separately (including all control variables), while Model 5 is the full model. The interaction models (Models 6 and 7) are presented in Figs. 1 and 2.

In line with the results of existing research and with hypothesis H1a, Model 1 (Table 2) showed that tenants were significantly more likely to have dissolved their union (AME = 0.013, *p * = 0.01) than couples who were living in joint homeownership (reference). We find mixed support for hypothesis H1b: On the one hand, being in the *other* category (e.g., non-tenants living in a dwelling owned by a partner’s parents) was positively related to union dissolution (AME = 0.024, *p * = 0.01). On the other hand, couples who were living in a home owned by only one of the partners were not significantly more likely to separate than those who were living in a jointly owned home. This finding was robust even after the sole homeowner’s gender was taken into account. Model 2 shows the result for the operationalization including male and female sole homeowners as separate categories. Contrary to our expectations (hypothesis H1c), neither differed significantly from joint homeowners in their separation risks.^7^

Model 3 presents the results for housing affordability. As hypothesized (H3a), the results indicated that the couples in the high-residual-income group (i.e., those who had the most income left over after housing costs were deducted) were significantly less likely to separate than those in the medium group. When the reference category was changed to the high-residual-income group, it was revealed that this group also had a significantly lower risk of separation than the low-residual-income group (results not shown here).^8^

In Model 4, we included household density. We find no support for hypothesis H2a, as our results show that there was no significant difference in separation risks between couples in low-, medium- and high-density households.^9^ In the next step, we were interested in investigating whether the effect of density varied among couples with different levels of residual income (Fig. 1, Model 6) as suggested in hypothesis H2b.

The graph reveals that the interaction effect was insignificant for most categories: the predicted probability of union dissolution among couples living in a crowded household did not significantly differ by income group. (Only for the high-residual-income group did density make a difference: those couples who did not have any affordability problems were significantly less likely to separate if they lived in high-density households compared to those in medium-density households. It should, however, be noted that this group was small and therefore we refrain from making strong conclusions from this.) The results of Model 5 refer to the full model (without gender of sole homeowners). The findings for housing tenure status and housing affordability remained unchanged compared to Models 1 and 3, respectively. The AME for density further shrank and remained insignificant in the full model. This result might reflect the fact that homeowners usually live in larger dwellings than tenants. But in additional analyses on tenants only, the AME for density also did not reach statistical significance (results not shown here). In sum, we do not find any evidence for an effect of household density on union dissolution, contrary to our hypotheses (H2a and H2b). This contradicts the results of a recent study in the UK that found a positive association between density-related stress and union dissolution (Coulter and Thomas 2019). In order to make sure that this difference was not only due to variable measurement, we also used alternative operationalizations of household density. In our analyses using the log of the persons per room ratio, the positive and significant AME disappeared after taking into consideration housing tenure in the full model (not shown here). Also, the results for alternative ordinal measurement of household density with two or four categories did not differ significantly from those presented in Table 2.

Figure 2 presents the results for the interaction between housing affordability and settlement size (Model 7). We expect that the housing market should be less tight in rural than in urban areas, and that it should therefore be easier to find alternative living arrangements for each partner after a separation in rural areas. This effect should be strongest for those in the low-residual-income group. In Fig. 2, the reference category is *large settlement size* (> 100,000 inhabitants). The graph shows that in our data, there was no significant difference in the risk of union dissolution by settlement size, including for those in the low-income group. Thus, based on this (admittedly very rough) measure of the availability of affordable housing, we found no support for hypothesis H3b.

The results for the control variables were largely in line with our expectations and were robust across the models. The AMEs of the duration of the coresidential partnership were significant in most of the models. The findings further indicated that childlessness was positively associated with the risk of union dissolution, while being married had a negative AME. The number of children was found to be less relevant, as the estimates for having more than one child were insignificant. The positive and significant AMEs for region (eastern/western Germany) found in most of the models indicate that the stability levels of coresidential unions were lower in eastern than in western Germany.

### Sensitivity Analyses

In order to test whether our results are robust across different model specifications and samples, we performed some sensitivity analyses (Table 3). First, our sample is left-censored because coresidential couples that have separated before the onset of the pairfam study are not included. Therefore, there might be a positive selection of stable couples. In order to test for potential bias, we restricted the sample to couples who were living in a coresidential union for less than 6 years (5091 person-years, i.e., 31% of the original sample) excluding the overrepresented couples with long union durations. The results in Model 8 show that among couples with shorter relationships, sole homeownership was significantly associated with higher separation risks compared to joint homeowners, which was not the case in the full sample. However in the sample of couples with shorter relationship durations, the group of joint homeowners is considerably smaller than in the full sample (9.1% in the short-duration sample compared to 26.6% in the full sample). Couples who decide to jointly own property after living together for less than 6 years might be a selective group with very high relationship satisfaction—and thus lower separation risks.

In the second sensitivity check, we performed additional analyses that included *partnership satisfaction* as a control. In line with our argument in the theoretical section of this paper, we expected that partnership quality would be an important mediator in the effect of housing quality on separation risk. We excluded partnership satisfaction from our main analyses in order to avoid overcontrol bias, and because we are interested in the total effect. It should be noted, however, that partnership satisfaction can also be a confounder, because it is negatively related to the risk of separation, as well as to housing outcomes. For instance, the literature has shown that couples who report having high levels of relationship satisfaction are more likely to become homeowners (Brüderl and Kalter 2001). Conversely, it seems plausible that couples who report having low levels of partnership satisfaction are less likely to invest in an extensive search for better housing because they anticipate splitting up. In Model 9, we tested the alternative model specification including relationship satisfaction as a control variable. In line with theoretical expectations, the results show that respondents who were less satisfied with their partnership had higher separation risks, while those who were very satisfied with their partnership had lower dissolution risks. The AMEs of housing tenure and affordability shrink in size, which indicates that these effects are partly mediated by relationship satisfaction.

In our third sensitivity analysis, we test whether the housing variables were associated with the risk of union dissolution apart from the couples’ overall income situations. Model 10 includes the net household income in 100-euro increments as a control variable in order to account for the general socioeconomic situation of a couple. The effect of household income was insignificant in the model with the other control variables, while the AME of having a high residual income was significant in this specification as well. In other words, even after controlling for household income, housing affordability was statistically associated with the risk of separation.

## Discussion

Because housing markets in many western countries have undergone substantial changes in recent years (for example, house prices have generally increased following the 2007 financial crisis), it is important to improve our understanding of the effects of housing on partnerships. In this study, we investigated the relationship between couples’ housing conditions and their likelihood of union dissolution. Prior research on the association between couples’ socioeconomic characteristics and their risk of separation generated mixed findings. We argued that housing is a neglected component of a couple’s living standard that is related to the couple’s well-being and relationship stability. We focused on three aspects of housing: housing tenure, housing affordability and household density. Overall, our findings imply that housing affordability and housing tenure status are important factors in relationship outcomes and should therefore be taken into consideration in future studies. We did not, however, find a significant association between household density and union dissolution.

The results of this study contribute to the literature in several ways. First, the finding that couples with higher residual income had a lower risk of union dissolution is in line with the few studies on this topic (Lauster 2008; Coulter and Thomas 2019). This implies that housing costs might lead to tensions and conflicts between partners and thus increases the risk of separation. To ensure that we have not confounded the effects of housing affordability and socioeconomic status, we performed a simple sensitivity analysis with absolute household income as a control variable. The effect of housing affordability remained stable in this additional analysis, which implies that housing costs have an independent effect on partnership stability regardless of overall income. These complex associations should be further analyzed in future studies to determine whether similar effects are found in other settings.

Second, in line with previous results and our own hypothesis (H1a), we found that homeowners had lower dissolution risks than tenants. In addition to examining the usual homeowner–tenant dichotomy, we were able to differentiate between joint homeowners and sole homeowners (i.e., couples in which only one partner owns the dwelling). The separation risks of the group of sole homeowners were not found to differ significantly from those of the group of joint homeowners. This is an interesting finding. We expected to find that only joint homeownership was indicative of a mutual investment in the relationship and was thus associated with union stability. A number of studies that investigated this topic used an indicator that controlled only for joint homeownership versus all other housing tenure types. However, our results for sole homeowners suggest that they do not differ significantly from joint homeowners. Thus, in future studies, sole homeowners should not be lumped together in a single group with non-owners. Yet the interpretation of this finding is not straightforward. On the one hand, if one partner in a couple was a homeowner before the relationship started, having the other partner move in might signal a strong commitment to the relationship. On the other hand, it is possible that only one partner is registered as the homeowner for practical reasons, but both partners have invested in the home. The pattern for sole homeownership was the same for men and women, i.e., our hypothesis H1c, that sole homeownership informs us about the gendered independence structure within a couple, was not supported in our data.

Moreover, the dissolution risks of couples who were living in the property of a third party were found to be even higher than those of tenants. This finding might reflect the likelihood that moving into a home owned by one of the partner’s parents represents a transitory housing situation. It should be noted, however, that the share of couples who were in the *other non-tenant* group was small (5% in our sample). Additional research is needed to further investigate the underlying mechanisms of these different ownership types and their relationship to union stability.

Third, we did not find an association between household density and union dissolution. We expected to find that reduced private space was negatively associated with a person’s emotional well-being and was thus related to decreased relationship stability (H2a), but this idea was not supported in the analyses. Our findings are in line with existing evidence in Finland (Jalovaara 2002) and Luxembourg (van Damme 2019). However, other studies do find a positive association between room stress and separation (Coulter and Thomas 2019 in the UK; Gerber and Zavisca 2015 in Russia). Future research should seek to replicate our results by investigating the union dissolution patterns of couples in crowded households in other countries. This would help to identify settings in which household density is associated with union dissolution. Moreover, in other country contexts, it might be possible to analyze separately the effects of sharing a household with people other than the couple’s own children; in the German setting, the share of households in this category is very small.

At first glance, the AMEs resulting from our models seem to be small in size. For example in Model 5, the average predicted probability of experiencing a union dissolution since the last wave among the couples in the high-residual-income group was 0.7 percentage points lower than that of the couples in the medium-residual-income group. However, within a single year, a separation is a rare event. In the 7-year period under study, only 11.6% of the partnerships in our sample broke up, which indicates that the probability of experiencing the separation of a coresidential union is generally low. A comparison with the AME of being married might help to put our results into perspective: the predicted probability of separation for unmarried couples was, on average, 2.2 percentage points lower than that of married couples (Model 5). This means that the AME for the high-residual-income group was about one-third the size of the AME for the married group. Given the relevance of marriage for couple stability, this finding indicates that the association between housing affordability and the risk of union dissolution was non-negligible. In sum, and in line with the results of prior research, we found that demographic characteristics seem to explain the largest share of the probability of union dissolution among the explanatory variables included in the regression models.

Most of the results we have presented here refer to the average couple; i.e., we did not delve into important differences that might exist across partnerships and within couples. Existing research has, for instance, shown that partners do not necessarily agree on the intention to move (Coulter et al. 2012); and it is also likely that partners sometimes differ in their evaluations of housing conditions. Moreover, the process of union dissolution is complex and may follow heterogeneous patterns. It could be argued that in addition to looking at the impact of housing affordability on the risk of separation, researchers should account more explicitly for couple dynamics and family stratification. Some of these dynamics may include the relative income distribution within each couple and partners’ perceptions of the fairness of their respective contributions to household expenses—aspects that might affect both housing decisions and separation. However, such information was available for only a subsample of couples in pairfam and therefore, we did not consider this in our analyses.^10^ Moreover, future studies should further investigate the role of the availability of alternative dwellings. It is possible that individuals in the low-residual-income group in particular would have difficulties finding affordable housing after a union dissolution, and that such concerns might discourage them from separating. In our analyses, we could not find such an effect. However, we used settlement size as a proxy for the availability of affordable housing after separation. Future research should include more explicit measures to account for access to alternative housing.

A number of other aspects of the association between housing characteristics and the risk of separation could not be addressed in this study. Although we were focusing on objective housing indicators in our study, it seems plausible that the effect of housing varies; indeed, it has been shown that the perception of crowding differs across cultural settings (Kaya and Weber 2003; Palisi 1984) and across socioeconomic groups (Hu and Coulter 2017). Therefore, future studies should include subjective measures of crowding and housing cost burdens. Such subjective measures would also allow us to distinguish between those couples who prefer living in a dense home over living in a spacious place, or those who are willing to pay higher housing costs due to a preference for higher-amenity homes compared to those who prefer spending less on housing but more on other goods and services. In addition, the use of more precise indicators of housing quality or satisfaction with the housing situation and the neighborhood could provide us with important insights into the association between housing and the risk of union dissolution. From a theoretical point of view, we might expect to find that household density and housing affordability are related to psychological distress and thus increase the risk of union dissolution. In order to test whether the proposed mechanisms exist, it would be interesting to explicitly investigate the association between housing and stress. Unfortunately, the data collected in *pairfam* did not allow us to account for such factors.

## Figures and Tables

**Fig. 3:**
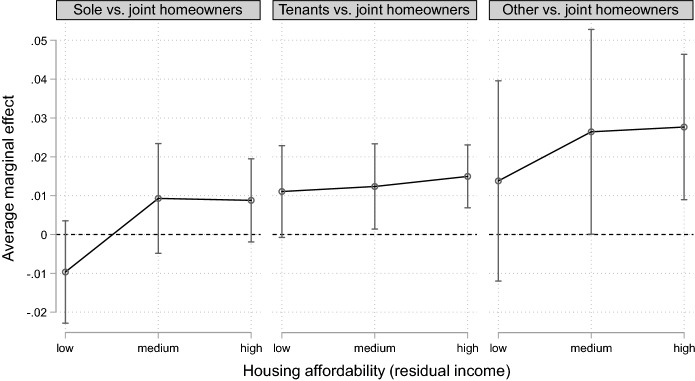
Interaction effect (Model 11). Average marginal effects of housing tenure on union dissolution for different levels of housing affordability (residual income after housing costs are deducted). Reference category: joint homeowners (dashed line). Source: pairfam Waves 1–7. Authors’ own calculation. Multiply imputed data. Control variables: duration of coresidential union, duration squared, age of female partner, partnership status, region, number of children in the household, household density. Average marginal effects and significance levels are presented in Table 6 in “Appendix”

## Appendix

See Tables 4, 5, 6 and Fig. 3.

**Table 4:** Discrete-time event-history models of union dissolution (average marginal effects). Complete cases. *Source*: Pairfam waves 1–7. Authors’ own calculation

	Model A1	Model A2	Model A3	Model A4	Model A5
Duration of coresidential union					
In years	− 0.000	− 0.000	− 0.000	− 0.000	− 0.000
In years squared	0.000	0.000	0.000	0.000	0.000
Age of female partner	− 0.002**	− 0.002**	− 0.002**	− 0.002**	− 0.001**
Children living in the household					
No children	0.003	0.003	0.006	0.002	0.005
1 Child	0	0	0	0	0
2 Children	0.003	0.003	0.001	0.001	0.002
3 Or more children	0.004	0.004	0.001	0.002	0.002
Partnership status					
Unmarried	0	0	0	0	0
Married	− 0.026**	− 0.026**	− 0.029**	− 0.029**	− 0.026**
Region					
Western Germany	0	0	0	0	0
Eastern Germany	0.002	0.002	0.002	0.002	0.001
Housing tenure					
Joint homeownership	0				0
Sole homeownership	0.006				0.007
Tenants	0.015**				0.014**
Other (e.g., living in parents’ property)	0.020**				0.020**
Housing tenure					
Joint homeownership		0			
Male sole homeowner		0.010*			
Female sole homeowner		0.000			
Tenants		0.015**			
Other (e.g., living in parents’ property)		0.020**			
Housing affordability					
Low residual income			0.002		0.001
Medium residual income			0		0
High residual income			− 0.006^#^		− 0.006^#^
Household density					
Low (< 1 persons/room)				− 0.005	− 0.001
Medium (1 person/room)				0	0
High (> 1 person/room)				0.002	0.001
Number of relationship-years Number of events	13,969 368	13,969 368	13,969 368	13,969 368	13,969 368

Complete cases analyses (without multiple imputation)

^#^*p* < 0.1, **p* < 0.05, ***p* < 0.01, ****p* < 0.001

**Table 5:** Discrete-time event-history models of union dissolution (odds ratios). *Source*: pairfam waves 1–7. Authors’ own calculation

	Model B1	Model B2	Model B3	Model B4	Model B5
Duration of coresidential union					
In years	0.966	0.966	0.958	0.959	0.968
In years squared	1.003*	1.003*	1.003*	1.003**	1.003*
Age of female partner	0.950***	0.950***	0.947***	0.943***	0.954***
Children living in the household					
No children	1.381*	1.381*	1.494**	1.367*	1.516**
1 Child	1	1	1	1	1
2 Children	1.236	1.235	1.161	1.188	1.215
3 Or more children	1.249	1.248	1.146	1.167	1.187
Partnership status					
Unmarried	1	1	1	1	1
Married	0.432***	0.432***	0.401***	0.400***	0.433***
Region					
Western Germany	1	1	1	1	1
Eastern Germany	1.257*	1.259*	1.243^#^	1.277*	1.223^#^
Housing tenure					
Joint homeownership	1				1
Sole homeownership	1.257				1.249
Tenants	1.899***				1.869***
Other (e.g., living in parents’ property)	2.594***				2.589***
Housing tenure					
Joint homeownership		1			
Male sole homeowner		1.320			
Female sole homeowner		1.148			
Tenants		1.902***			
Other (e.g., living in parents’ property)		2.599***			
Housing affordability					
Low residual income			1.001		0.959
Medium residual income			1		1
High residual income			0.747*		0.750*
Household density					
Low (< 1 persons/room)				0.918	1.043
Medium (1 person/room)				1	1
High (> 1 person/room)				1.148	1.100
Number of relationship-years Number of events	16,036 401	16,036 401	16,036 401	16,036 401	16,036 401

Multiply imputed data

^#^*p* < 0.1, **p* < 0.05, ***p* < 0.01, ****p* < 0.001. Multiply imputed data

**Table 6:** Interaction effects (models 6, 7 and 11). Discrete-time event-history models of union dissolution (average marginal effects). *Source*: Pairfam waves 1–7. Authors’ own calculation. Multiply imputed data. Control variables: duration of coresidential union, duration squared, age of female partner, partnership status, region, number of children in the household. In Model 6: also housing tenure. In Model 7: Also housing tenure and household density. In Model 11: also household density

	Housing affordability (residual income)		
	Low	Medium	High
*Model 6*			
Household density			
Low (< 1 persons/room)	− 0.001	− 0.003	0.002
Medium (1 person/room)	0	0	0
High (> 1 person/room)	0.011	− 0.012	− 0.015*
*Model 7*			
Settlement size			
> 100,000 inhabitants	0	0	0
20,000–100,000 inhabitants	− 0.006	0.003	0.004
< 20.000 inhabitants	− 0.004	0.003	0.003
*Model 11*			
Housing tenure			
Joint homeownership	0	0	0
Sole homeownership	− 0.010	0.009	0.007
Tenants	0.011^#^	0.012*	0.015**
Other (e.g., living in parents’ property)	0.014	0.027*	0.025**

^#^*p* < 0.1, **p* < 0.05, ***p* < 0.01, ****p* < 0.001

## Footnotes

1 As the number of multiple cohabitations of the same main respondent was small, we refrained from running multilevel analyses.

2 Moreover, we excluded those couples who reported a cohabitation break because if the partners did not share a dwelling, their housing conditions were less relevant for their partnership development.

3 We calculated an alternative measure of the person–room ratio in which we counted all household members as one person (instead of 0.5 of a person for each child under age 12). The use of this measure led to substantially the same results in our regression analyses (not shown here).

4 When we checked how high-density living was distributed among higher- and lower-income households, we found that high density was more common in households with below median income (9.2%) and less common in households with median income or higher (2.8%).

5 This correlation coefficient is calculated based on the complete case sample of our analyses.

6 When running regression models with other socioeconomic variables, such as partners’ education and labor force status, the effects of residual income turned insignificant; but the added variables themselves did not show strong and significant effects. We interpret this result as an overspecification of the model. Given our focus on housing characteristics, and in order to keep the models parsimonious, we decided to exclude the employment and education characteristics of the two partners from the models.

7 Changing the reference category, the difference between male and female homeowners was also insignificant (results not shown).

8 In order to determine whether the effect of housing affordability might differ according to housing tenure status, we performed an additional analysis. However, we found no indication of an interaction of housing costs and housing tenure on the risk of separation (see Fig. 3 in “Appendix”).

9 In the baseline model (not shown here) with only the two relationship duration variables and density, we did find a significantly increased dissolution risk among couples who were living in a low-density household (i.e., a household in which the average number of persons per rooms was smaller than one) compared to couples in dwellings with a medium level of density (reference category). However, this effect turned insignificant after including the other control variables.

10 Controlling for age and education homogamy did not change our results substantially.
