# Correction to: Housing Affordability, Housing Tenure Status and Household Density: Are Housing Characteristics Associated with Union Dissolution?

**DOI:** 10.1007/s10680-021-09587-z

**Published:** 2021-06-07

**Authors:** Sandra Krapf, Michael Wagner

**Affiliations:** 1 Mannheim Center for European Social Research, University of Mannheim, 68159 Mannheim, Germany; 2 Institute of Sociology and Social Psychology, University of Cologne, Albertus-Magnus-Platz, 50923 Cologne, Germany

## Correction to: European Journal of Population (2020) 36:735–764 https://doi.org/10.1007/s10680-019-09549-6

The article “Housing Affordability, Housing Tenure Status and Household Density: Are Housing Characteristics Associated with Union Dissolution?” written by Sandra Krapf and Michael Wagner, was originally published electronically on the publisher’s internet portal on 10 January 2020 without Open Access. With the authors’ decision to opt for Open Choice, the copyright of the article changed on 5 May 2021 to © The Author(s) 2020 and the article is forthwith distributed under a Creative Commons Attribution 4.0 International License, which permits use, sharing, adaptation, distribution and reproduction in any medium or format, as long as you give appropriate credit to the original author(s) and the source, provide a link to the Creative Commons licence and indicate if changes were made. The images or other third party material in this article are included in the article’s Creative Commons licence, unless indicated otherwise in a credit line to the material. If material is not included in the article’s Creative Commons licence and your intended use is not permitted by statutory regulation or exceeds the permitted use, you will need to obtain permission directly from the copyright holder. To view a copy of this licence, visit http://creativecommons.org/licenses/by/4.0/.

